# Use of supravital toluidine blue staining to improve the efficiency of fine-needle aspiration cytology reporting in comparison to papanicolaou stain

**DOI:** 10.12669/pjms.315.8411

**Published:** 2015

**Authors:** Kanwal Saba, Shahida Niazi, Mulazim Hussain Bukhari, Sardar Fakhar Imam

**Affiliations:** 1 Kanwal Saba, Department of Pathology and Medicine, Fatima Jinnah Medical College, Lahore, Pakistan; 2 Shahida Niazi, Dept. of Pathology, King Edward Medical University, Lahore, Pakistan; 3 Mulazim Hussain Bukhari, Department of Pathology and Medicine, Fatima Jinnah Medical College, Lahore, Pakistan; 4 Sardar Fakhar Imam, Department of Pathology and Medicine, Fatima Jinnah Medical College, Lahore, Pakistan

**Keywords:** FNAC, Toluidine stain smear, Papanicolaou smears, Supra vital stains

## Abstract

**Objective:**

To see the efficiency, adequacy and accuracy of toluidine blue stained smears of FNAC of Breast thyroid and salivary glands swelling with comparison to conventional stained FNAC smears with Papanicolaou.

**Methods:**

A total of 114 aspirates from various sites were included in the study. The smears were stained with toluidine blue and conventional Papanicolaou stain and the cytomorphology of both the smears were compared. The values were tabulated and statistical tests of significance was applied.

**Results:**

Of the 114 aspirates included in our study the diagnostic accuracy by using papanicolaou was 78%, while it was upto 100% with supravital toluidine blue stained smears. The percentage of inadequacy was reduced to just 25%. The observations were statistically significant. Breast 37/48 (77%) and Salivary glands 11/48 (23%) respectively. The most commonly used categorization of a five-tier system was used for reporting of breast cytology, with categories ranging from insufficient materials (C1), benign (C2), atypical (C3), suspicious of malignancy (C4), or (C5) frankly malignant. Most of breast lesions were benign 25 (67.56%). There were only 9 (24.32%) malignant cases followed by 2 cases of C-4 and one case of C-3. Benign thyroid lesion were more frequent comprising of 51 (72.27%) cases. One case (1.5%) of papillary carcinoma was found while 13 case were follicular lesions. There were 4 (36.4%) cases of pleomorphic adenoma and 3 (27.3) cases of non-specific sialadenitis. There was one case (9%) of each lesion for mucoepidermoid carcinoma, adenoidcytic carcinoma and benign cyst.

**Conclusion:**

Toluidine blue stained study of FNAC improves the diagnostic accuracy by minimizing the smearing and drying artifact, loss of cell sample during fixation and staining which influences the diagnostic accuracy.

## INTRODUCTION

Fine needle aspiration cytology (FNAC), is an essential diagnostic technique, used to investigate superficial and deep swellings. In this procedure, a thin, hollow needle is inserted into the swellings for aspiration of cells. The smear is stained with different stains for microscopic examination to reach a proper diagnosis.^1^,^2^

This procedure provides an early, quick and same day information to the surgeon about the type of lesion he is dealing with. Surgeons prefer to get information about their patients before performing the biopsy or surgery because compared to a biopsy report which is usually available in 2-4 days, the FNAC report is prepared within few hours of sampling.^2^

The success of FNAC depends on four fundamental requirements (a) samples must be representative of the lesion investigated, (b) samples must be adequate in terms of cells and other tissue components, (c) samples must be correctly smeared, (d) samples must be properly processed and stained and (e) reporting and diagnosis by an expert in cytology.^3^

The diagnostic accuracy of FNAC depends on adequacy of sample, representativeness of the sample, and good cytomorphological detail without much artifactual distortion. Several authors have studied the immediate cytological evaluation using rapid stains to assess the sample adequacy and to improve the diagnostic accuracy.^4^-^6^

The non-diagnostic results of cytological smears are not due to examining the smears by inexperienced persons but also due to poor sample collection and preparation. Improving the quality of cytological submissions will maximize the likelihood of a meaningful cytological description and a more accurate cytological diagnosis.^7^-^9^

There are many procedures for accelerated diagnosis of breast swellings, thyroid nodules, lymph nodes, liver masses, subcutaneous swellings and swellings of the oral cavities, but FNAC is a relatively simple, accurate, a traumatic, economical and complication free technique for the evaluation of these lesions. One of the main advantages of FNAC is that it can be easily repeated if an adequate aspirate is not obtained.^10^-^17^

Toluidine blue is supra-vital stain that accentuates good nuclear detail and enables a three dimensional view of cells in a wet mount film. It is easily available, very cheap, cost effective and used for quick reporting. It also permits preservation of cytological material by destaining and restaining with permanent stains. It helps to obtain sufficient cellularity in less cellular fibrotic lesions. It is also used to assess adequacy of samples especially for deep seated lesions and minimizes false negative results. The cytomorphology is well appreciated in wet mount study. It can be used for intra operative cytodiagnosis as an adjunct to frozen section diagnosis. It improves the diagnostic accuracy of conventional FNAC’s in its different lesions.^18^-^22^

This study was conducted to see the efficiency of Supravital Toluidine Blue staining in comparison to Papanicolaou stain on FNAC in the diagnosis of breast and salivary glands lesions.

## METHODS

This cross sectional study was conducted in the Department of Pathology King Edward Medical University (a tertiary care hospital) with written consent of participants after approval from IRB from August to December, 2014. A total of 114 consecutive purposive sampling on palpable breast lumps and salivary glands swellings, referred from the Outpatient Department for FNAC were included in this study. We used 0.5% toluidine blue to stain FNAC slides for the evaluation of smear adequacy and morphology. The toluidine blue-stained slides were compared with fresh wet fixed smears stained with Papanicolaou staining. The data was entered in SPSS version 22 and accuracy was calculated by 2×2 Table.

## RESULTS

**Fig.1:**
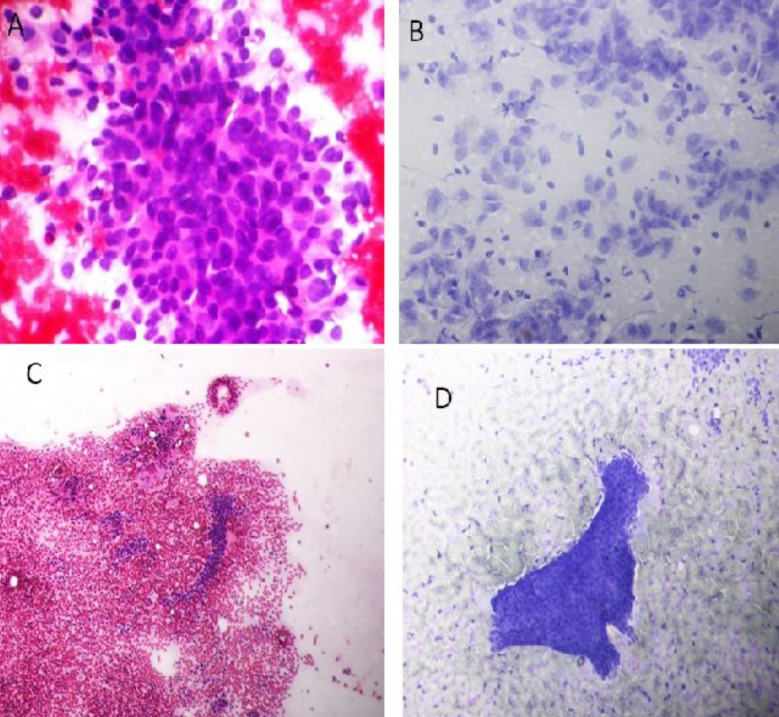
Photomicrograph of FNAC of Lump Breast, A: showing morphology of C-5 (40x PAP) with no cohesive sheets of pleomorphic cells (Ductal Carcinoma), B:showing morphology of C-5 (40x TBS) with non-cohesive sheets of pleomorphic cells (Ductal Carcinoma), C: showing morphology of C-2 (20x PAP) with tightly cohesive sheets of uniformly looking cells and naked nuclei of fibroblast (fibroadenoma) and D: showing morphology of C-2 (20x TBS) with tightly cohesive sheets of uniformly looking cells and naked nuclei of fibroblast (fibroadenoma).

FNAC was performed in 114 patients. The overall mean age was 32.08±10.54 years. There were 37 cases of breast lesions which presented for FNAC. The five tier system was used for reporting of breast cytology, with categories ranging from insufficient materials (C1), benign (C2), atypical (C3), suspicious of malignancy (C4), or(C5) frankly malignant (Fig.9-10). Most of breast lesions were benign, comprising 25 (67.56%) cases. There were only 9 (24.32%) malignant cases followed by 2 cases of C-4 and one case of C-3 (Table-I and Fig.1-4).

**Table-I:** Frequency of breast lesions on fine needle aspiration cytology (n=38).

Category	Description	Frequency	%age
C-2	Benign	25	67.56
C-3	Atypical probably benign	1	2.7
C-4	Atypical probably Malignant	2	5.4
C-5	Malignant	9	24.32
Total		37	

**Fig.2:**
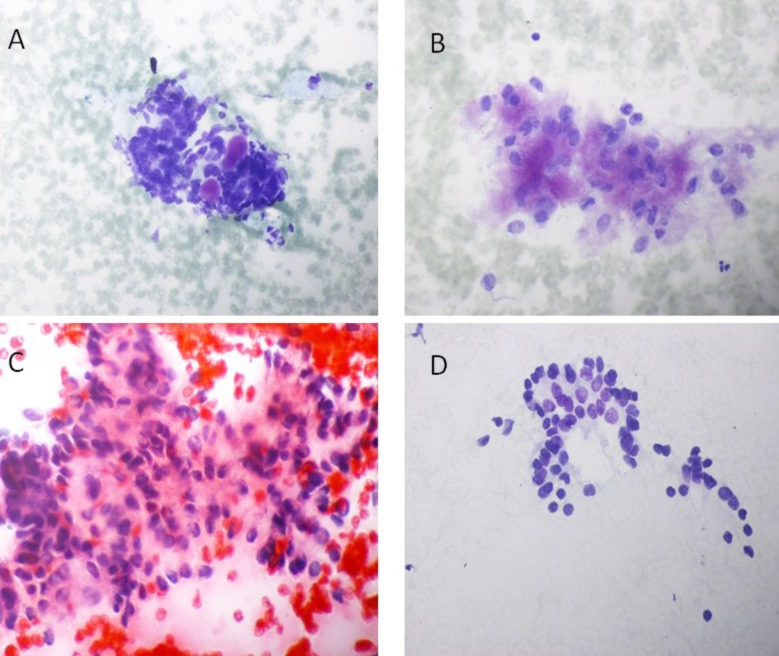
Photomicrograph of FNAC of A: Submandibular gland swelling showing morphology of (20x TBS) small cells with bland nuclear features forming a pseudoglandular space containing a homogeneous, metachromatically staining hyaline globule (adenoid cystic carcinoma), B: Parotid swelling showing morphology (20x TBS) of clusters of benign epithelial cells with blue myxoid matrix (Pleomorphic adenoma), C: Parotid swelling showing morphology (20x PAP) of clusters of benign epithelial and my epithelial cells with blue myxoid matrix (Pleomorphic adenoma) and D: swelling thyroids

Eleven cases of salivary gland swellings presented for fine needle aspiration. Among these 7 (63.6) were of submandibular glands and 4 (36.4%) were from parotid glands. There were 4 (36.4%) cases of pleomorphic adenoma and 3 (27.3%) cases of non-specific sialadenitis. There was one case (9%), each of mucoepidermoid carcinoma, adenoid cystic carcinoma and benign cyst. (Table-III and Fig.4).

**Table-II:** Frequency of various thyroid categories diagnosed on FNAC (n=66).

Category	Description	Frequency	Percent
C-I	Inadequate or Non-diagnostic	0	0
C-II	Benign follicular nodule	51	72.27
C-III	Cytological atypia of undetermined significance	1	1.5
C-VI	Follicular neoplasm or suspicious for a follicular neoplasm and specify if hürthle cell (oncocytic) type	13	19.7
C-V	Suspicious for Malignancy	0	0
C-VI	Malignantthyroid carcinoma other than follicular	1	1.5
Total		66	100

***Note:*** The above “The Bethesda System for Reporting Thyroid Cytopathology”: Recommended Diagnostic Categories used for reporting of FNAC.

**Table-III:** Distribution of salivary gland lesions presented for FNAC.

Pathology	Frequency	Percent
Pleomorphic adenoma	4	36.4
Non-specific Inflammation	3	27.2
Mucoepidermoid Carcinoma	1	9.1
Adenoid Cystic Carcinoma	1	9.1
Benign Cyst	1	9.1
Squamous Cell Carcinoma	1	9.1
Total	11	

Solitary thyroid nodules were most frequent lesions in thyroid patients and were seen in 46 (69.7%) cases Bathesda system was used. Fourteen cases of multinodular goiters (21.2%) and diffusely enlarged thyroid gland (6 cases 9.1%) also presented for FNACs. Benign thyroid lesions were more frequent comprising of 51 (72.27%) cases. One case (1.5%) of papillary carcinoma was found while 13 case were of follicular lesions. One case (1.5%) was of undetermined significance. Sixty five cases presented with thyroid scan for cold nodules while only one case was of hot nodules. (Table-II and Fig.5-8).

Toluidine blue staining (TBS) was adequate for all patients and were re-pricked if there was any unsatisfactory smearon site. There were 24% non-diagnostic smears for Papanicolaou stains. No technician was required for FNAC smears which were stained with TBS while a technical staff was required for FNAC smears which were stained with Pap stains. Similarly no alcohol was needed for fixation for TBS methods and no Laboratory infrastructure was used for this procedure. Per test cost was Rs.95.00 for TBS technique and Rs. 435.00 for PAP technique. Rapid stain study with TBS yielded 100% accuracy in all cases of aspirates whereas PAP stain study gave 78% accuracy. The rate of efficiency for onsite reporting for TBS was 100% while for PAP no onsite report was sent for reporting of fine needle aspiration. All reports were provisionally issued to patients after getting their consent within 3 minutes of their procedure while all smears were sent to laboratories for staining with Papanicolaou stains because full laboratory infrastructure was required for this technique. The staining ability of TBS was as good as with PAP staining. The staining intensity was also at par for both staining procedures. All 114 semars were permanently preserved after TBS and PAP staining. The cellular and nuclear detail was good for TBS while excellent for Pap staining. (Table IV & V)

**Table-IV:** Calculation of cost per test for both staining Procedures.

	Smear adequate	Non-diagnostic	Percentage of adequacy	Efficient Reports on site	Accuracy
Toluidine Blue staining	114	0	100%	100%	100%
Papanicolaou stain	90	24	78%	0	78%

**Table-V:** Reporting time and Qualitative analysis for both staining technique.

Stains	Procedure site	Frequency	Percent	Staining time Minutes	Report issuing site
TBS	Onsite	114	100.0	3.00	On site in Procedure room
	Not onsite	0	0.00		
Pap	Onsite	0	0.00	30.00	In the Laboratory after 24 hours
	Not onsite	114	100.0		
Stains	Staining ability	Staining intensity	Permanent smearing	Cell membrane and cytoplasm	Nuclei detail
TBS	Good	Good	Good	Good	Good
PAP	Good	Good	Good	Good	Good

## DISCUSSION

We have evaluated the utility of TBS smears in comparison to PAP stained smears and use of such toluidine rapid staining to improve the diagnostic yield. This study was designed to evaluate wet mount smears stained with toluidine blue to improve the efficiency of FNAC smears by comparing with Papanicolaou stained smears, and further to assess the reliability and accuracy of toluidine blue when compared to Pap stained FNAC smears.

Aspirate from benign breast lesions especially fibroadenoma showed uniform round and oval cells with scanty to moderate pale pink cytoplasm, round to oval uniform nucleus and granular chromatin with many bare ovoid nuclei. Fibrocystic lesions showed benign duct epithelial cells, cyst macrophages-large having round cells with vacuolated cytoplasm and round nucleus, eosinophilic secretions and few spindle cells entrapped within fat cell clusters. Aspirate from fat necrosis slowed many fat globules entangled in pale pink granular necrotic material and few macrophages. Malignant lesions showed pleomorphic cells with scant to moderate blue cytoplasm, large darkly stained nucleus with smudged chromatin and inconspicuous nucleoli and necrotic material in the background.^25^

FNAC is commonly used in the diagnosis of lump breast because it is an excellent, safe, and economical diagnostic modality on-site procedure for quick reporting with minimal inexpensive equipment.^15^,^26^ Most of breast FNAC were benign (67.56% cases). While malignant lesions were less common as compared to benign lesion. There were only 24.32% malignant cases followed by 2 cases of C-4 (suspicious for malignancy) and one case of C-3 (Suspicious for benignancy).

In a similar study by Bukhari et al in 2011, there were 20% cases of benign inflammatory lesions of acute and chronic mastitis, therefore our findings are fairly consistent with our own previous studies^15^,^27^ and also with international studies.^28^

The advantage of salivary gland FNAC is widely accepted due to the fact that it is easy to perform, minimally invasive, rapid smear evaluation, and repeatable to obtain more tissue for diagnosis or special studies. It is generally agreed by many cytopathologists that FNA of salivary glands is a good diagnostic test with reasonable sensitivity and specificity, ranging from 60% to 100% and 90% to 100%, respectively.^29^

Salivary gland, benign lesions showed round to oval cells with distinct cell membrane, moderate pink cytoplasm, plump ovoid nucleus with granular chromatin and uniform spindle cells. Malignant lesions showed sheets of pleomorphic cells with scanty pink cytoplasm, large anisokaryotic hyper chromatic nucleus.

There were 66 cases of thyroid swellings who gave consent for this FNAC study. We used the Bethesda Reporting System for reporting FNAC of thyroid nodules according to the literature and used six diagnostic categories to standardize communication of thyroid fine-needle aspiration (FNA) interpretations between clinicians and cytopathologists.^30^

Benign thyroid lesion (C-2) were more frequent comprising of 51 (72.27%) cases. One case (1.5%) of papillary carcinoma (C-6) was found while 13 cases were of follicular lesions (C-4). One case (1.5%) was of undetermined significance (C-3). There were 98.5% cases presented with thyroid scan for cold nodules while only one case (1.5%) was of hot nodule. The findings are different from Basharat et al.,^31^ because of the use of a different reporting system. In their report, there were 80% patients who had cold nodules on thyroid scan while 20 patients had hot nodules on thyroid scan. Our findings are also not consistent with Al-Sindi et al, who also used different reporting system than “TBSRTC”. The difference may be due to sample size in both local studies. Our findings are also not consistent with Park et al who used same reporting system.^32^

Their findings showed that the percentage of FNAC diagnoses in category II in our study (40.6%) was lower than that in other studies. While the percentage of FNAC diagnoses in their study with categories V and VI was 19.3% and 17.3%, respectively which was higher than that of our findings. There was also a significant difference in category III and IV. One possible reason for these differences may be that their hospital was a referral hospital for thyroid surgery, therefore the patients who were suspected of having thyroid malignancy were referred here where they conducted their study.

There were 11 cases of salivary gland swellings who presented for fine needle aspiration. There was one case (9%) each for mucoepidermoid carcinoma, adenoid cystic carcinoma and benign cyst. The findings are consistent with literature, where it was found that benign tumors constitutes the majority of salivary gland tumors, accounting for about 85%,63% and 14% in the parotid, submandibular, and sublingual glands, respectively. However in our study the frequency of parotid gland lesions was low as compared to that mentioned in literature.^29^

The study findings are also in accordance with a locally conducted study, where they found that the Parotid gland was involved in 68%, submandibular gland in 30%. There were 14% cases of non-neoplastic lesions and 86% cases of neoplastic lesions on biopsy. The difference may be due small number of our cases as compared to Ashraf et al who conducted study on 100 cases.^33^

Pleomorphic adenoma was the most common benign salivary gland lesion seen in our study (36.4%) This finding is supported by many previous studies.^25^-^34^

Toluidine blue staining (TBS) was performed onsite to see the adequacy and efficiency of smears. Wet mount smears were evaluated immediately under microscope in procedure room. The staining took 3 minutes and immediate assessment was made. The Pap smears were sent to the laboratory and were stained with the help of technicians and technologists. The adequacy could not be assessed onsite.

TBS smears were adequate for 48 patients as were re-pricked if there were any hemorrhagic or acellular or inadequate smear on site within 3 minutes. When TBS smears were compared next day with Pap stained smears reports, there were 25% inadequate smears on Papanicolaou stains. This difference was statistically highly significant p= is 0.000).

Our study employed TBS, which is a cheap and easily available supravital stain and results from our study are promising in terms of adequacy assessment as compared to conventional Pap stained smears. The accuracy for TBS was 100% while for Pap was 78%. The rate of efficiency for onsite reporting for TBS was 100% while for Pap no onsite report was sent for reporting of fine needle aspiration.

The TBS for rapid staining in assessment of aspiration adequacy was detected as 100% improvement in the efficiency of FNAC and these on-site staining should dramatically reduce the rate of failures and thus enable same-day reporting and repeat sampling. These observations are similar with the other investigators conclusion.^35^-^38^

The staining ability of TBS was as good as with Pap staining. The staining intensity was also at par for both staining procedures. All 200 stains were permanently preserved after TBS and PAP staining. The cellular and nuclear detail was good for TBS while excellent for PAP staining. Cytological morphology on FNAC and differentiation were excellent with TBS. Nuclear and nucleolar details were as good as PAP smears. The TBS technique decreased the need for revisiting for re-aspiration in 100% cases as compared to PAP and increased the laboratory turnover as well as the diagnostic yield to 100%. These findings are comparable with other studies.^9^,^38^

## CONCLUSION

Supravital stained rapid FNAC with TBS is useful as a simple reliable, cost effective rapid staining method. It helps to obtain sufficient cellularity in less cellular lesions. It is also used to assess adequacy of sample especially for deep seated lesions and minimizes false negative results.
